# Knowledge and Perception Toward Neuromodulation Devices Among Medical Students at Umm Al-Qura University

**DOI:** 10.7759/cureus.45256

**Published:** 2023-09-14

**Authors:** Taif F Alqahtani, Fadi S Althobaiti, Afnan J Alalyani, Lamyaa Zamzami, Mustafa Madani, Yazeed A Almalki, Abdullah Almogbil, Samah Y Labban

**Affiliations:** 1 Neurology, Umm Al-Qura University, Makkah, SAU; 2 Physiology, Umm Al-Qura University, Makkah, SAU

**Keywords:** psychiatric disorders, uqu, medical education, students’ knowledge, neurologic disorders, neuromodulation

## Abstract

Introduction

Neurologic and psychiatric disorders affect many people worldwide and are crucial to medical care. It is crucial to note that primary care doctors initially evaluate patients who will eventually require neuromodulation (NM) therapy. There is a growing concern about the extent of medical students' knowledge regarding NM therapy. Insufficient education and limited exposure of future doctors to different treatment approaches can limit their ability to refer patients promptly and appropriately, thereby impeding access to necessary treatment.

Methods

The study employed a non-probability stratified snowball sampling technique to recruit participants. The population consisted of undergraduate medical students (excluding interns) at Umm Al-Qura University (UQU) in Saudi Arabia. Data collection was conducted through an online questionnaire.

Results

The sample comprised 301 medical students, with an average age of 21.62±1.54 years (ranging from 18 to 25). The majority were female (65.1%), and in the clinical years (57.8%), a considerable portion of respondents (57.5%) lacked awareness that NM devices are approved by the FDA for treatment. Both pre-clinical and clinical-year students exhibited insufficient knowledge (91.3% and 91.4%, respectively). Females showed a higher proportion (95.9%) of poor attitudes toward NM compared to males (83.8%). Notably, preclinical students showed a higher level of knowledge (11.0%) compared to clinical students (6.3%).

Conclusions

The study revealed a significant lack of knowledge among medical students regarding NM devices. This is concerning given the growing prevalence of NM devices in clinical practice. To ensure optimal patient care, it is crucial to provide comprehensive education on NM devices to medical students.

## Introduction

Neurologic and psychiatric disorders affect millions of people worldwide, and in medical care, they are regularly encountered and typically linked with poor outcomes [1]. It is, therefore, important to note that inadequate education or exposure of future doctors to treatment modalities may adversely affect their ability to make timely and appropriate referrals to ultimately provide patients with the treatment they require.

Neuromodulation (NM) is a fast-growing field of neurotechnology that offers modulative effects on the central or peripheral nervous systems. NM involves the placement of electrodes into precise neural targets with the purpose of administering physical energy and modifying deviant brain activity. Consequently, suppressing a particular diseased area can restore a healthy neural system [2]. As a result of recent advancements in neurotechnology, neuroimaging, and neurocircuitry, the effectiveness of neurostimulation devices has continued to rise over time to treat an expanding variety of neurologic and psychiatric issues [3].

Vagal nerve stimulation and the responsive neurostimulation system are two NM devices that have been approved by the FDA for treating epilepsy [4-7]. Nowadays, NM is the standard of care for treating drug-resistant Parkinson's disease as a more effective and less-complication-associated alternative to ablative surgical procedures [8]. Other diseases have received FDA approval, such as essential tremors, dystonia, and obsessive-compulsive disorder. Similarly, intermittent theta-burst stimulation for the treatment of resistant depression has also received FDA approval [8].

In addition, NM has become a gold-standard therapy in motor circuit disorders due to advances made over the last two decades; several clinical trials have also studied its efficacy in other emerging nonmotor indications [9,10]. Despite the growing number of patients who undergo NM yearly, this therapeutic modality is still underutilized for a variety of different reasons [8].

Besides technical and clinical challenges, patients’ expectations must be considered, in addition to the knowledge level among medical students and general practitioners. Most patients who will be referred to NM at some point during their disorder will be seen first by primary care physicians.

Medical students’ knowledge of NM therapy has been a concern. In a study involving students at Hannover Medical School who were asked to fill out a questionnaire that included questions about DBS throughout their pre-clinical study period and in their last year of study, they showed partial awareness of DBS in the preclinical years, but as they continued their studies, their knowledge expanded in the following years [11].

On the contrary, a study at Virginia Tech Carilion School of Medicine, which involved 65 medical students, found no differences between the surveyed pre-clinical and clinical students regarding knowledge of DBS [12].

Currently, there are no specific guidelines for medical education in NM; exposure to these devices is likely important in improving medical students' knowledge and comfort levels. In another study conducted to assess the knowledge level regarding electroconvulsive therapy (ECT), medical students who had been exposed to such a treatment modality were proven to be more knowledgeable [13]. Similarly, the use of video-based educational materials resulted in a measurable improvement in students’ knowledge and attitudes regarding ECT [14]. These studies suggest that the direct exposure component of medical education may be important.

Little is known about the knowledge level of NM devices among medical students. To obtain a deep insight into this area and to identify in which aspects medical students’ knowledge is lacking, this study aims to explore the current knowledge and attitude of Umm Al-Qura University (UQU) medical students toward NM devices as a treatment modality and compare clinical and pre-clinical years.

## Materials and methods

Study design

While UQU's medical curriculum includes some exposure to relevant topics, such as neuroscience and behavior in the third year (pre-clinical) with seven weeks of basic science, and clinical neurology in the fourth year (clinical) with two weeks of theoretical as a part of other topics in medicine followed by two weeks for neurology clinical placements, it should be noted that NM devices are not comprehensively covered in all years. These topics may only be briefly mentioned rather than extensively taught in the curriculum. Psychiatry is covered in the fifth year (clinical) under the name of mental health, with a total of 14 theoretical lectures and only one week of clinical placement. A descriptive cross-sectional study was undertaken to evaluate the knowledge and perception pertaining to NM devices among medical students at the UQU in Saudi Arabia, spanning the period from February 2023 to April 2023. Employing a non-probability stratified snowball sampling methodology, participants were recruited. The data collection process employed an online questionnaire, which was subsequently integrated into Google Forms (Google LLC, Mountain View, California, United States). This instrument was electronically administered to the respondents, primarily through the utilization of social media platforms, with a significant emphasis on WhatsApp (Meta Platforms, Menlo Park, California, United States) as the primary medium of dissemination. In order to ensure a precise and targeted allocation of the questionnaire, the WhatsApp contact details of the students were collected with the assistance of data collectors from each academic batch, who collaborated with the authors in the data collection phase.

Study population

The study's target population was all undergraduate medical students who gave their consent to participate in the survey at UQU in Makkah, Saudi Arabia. Interns were excluded.

Sampling methodology and data collection

The sample size was calculated using OpenEpi version 3.0. The university's academic affairs department estimated the number of all academic year medical students at around 1,400. The calculation of the sample size was based on a 95% CI with 50% frequency and a design effect of 1. Three hundred medical students were required at least. The sample was distributed equally among the academic years. As for the study tool, an organized, self-administered online questionnaire was used to collect data.

The questionnaire was adopted from another survey study with some modifications to cover NM devices in general [12]. The survey consisted of a 25-item questionnaire, including a demographic section, a knowledge inventory to assess attitudes toward NM, and a self-perceived education on NM. Additionally, there is an independent item, a self-assessment of knowledge. The questions on knowledge and self-perception of education were multiple choice (yes = 1, I don’t know = 0, and no = -1). Whereas attitude responses were based on a five-point Likert scale, scores of 1, 0.5, 0, -0.5, and -1 were assigned for each of the answers. Finally, the sum of knowledge and attitude questions was graded as "good" and "positive," respectively, if the score was 60% or higher; participants who had a score of more than 5 were considered to have good knowledge, and more than 4 were considered to have a good attitude. Self-assessment of knowledge was measured by asking students to judge their understanding of NM on a seven-point scale (1 = vague understanding to 7 = thorough understanding).

Data analysis

A nonparametric, univariate analysis was performed. A descriptive analysis was conducted based on frequencies and percentages for categorical variables. A Chi-square test was used to assess the associations between the variables. Significance was assumed at p<0.05 for all tests. The data were analyzed using BlueSky Statistics version 10.2.1 (BlueSky Statistics, LLC, Chicago, Illinois, United States).

Ethical part and confidentiality

Participation in the study was entirely voluntary, and consent was obtained from each participant before their involvement in the survey. The survey provided a comprehensive overview of the study's objectives, which were presented to all participants. In order to protect the privacy of the participants, their identities were carefully kept confidential and anonymous, and only the research team had access to the collected responses. Additionally, it's worth mentioning that this study received ethical approval from the Biomedical Ethics Committee of UQU under approval number HAPO-02-K-012-2023-02-1429.

## Results

A total of 301 study participants were included in this study according to the inclusion and exclusion criteria; none were excluded. Table 1 represents demographic information about the sample population. The sample consisted of 301 medical students, with a mean age of 21.62±1.54 (range 18-25 years). The majority of the sample was female (65.1%), and most participants (57.8%) were in the clinical years of the study (years 4, 5, and 6). Regarding the intended medical specialties of the participants in the seven specialties listed, medicine was the most popular choice among the participants (16.0%), followed by general surgery (13.6%). Notably, more than 30% of the participants were undecided about their intended specialty. In addition, upon asking participants whether they have a family member who had been treated with NM devices, most of the respondents (79.7%) answered "no," 16.9% did not know, and only 3.3% answered "yes."

**Table 1:** Sociodemographic characteristics of the study participants NM: neuromodulation

Demographic characteristics (n=301)		
Age (years)	Range	18 to 25
	Mean	21.62 ± 1.54
Characteristic		(%)
Gender	Female	65.1
	Male	34.9
Year	Preclinical	42.2
	Clinical	57.8
Intended specialty	Emergency medicine	6.3
	General surgery	13.6
	Medical Sub-specialty	10.3
	Medicine	16
	Obstetrics and gynecology	3.3
	Psychiatry/Neurology	8.3
	Surgical sub-specialty	11
	Undecided	31.2
Family member treated with NM	Yes	3.3
	No	79.7
	I don't know	16.9

Tables 2-5 represent the results on knowledge inventory, self-perception of knowledge, attitude, and self-perception of education toward NM. The knowledge inventory results indicate that a significant proportion of respondents do not know that NM devices are an FDA-approved treatment (173, 57.5%). The utility of NM in treating movement disorders was agreed upon by 43.2%, compared to its utility in treating psychiatric disorders (37.9%). Neurologists and neurosurgeons were identified as being involved in administering NM by 43.5% and 41.2%, respectively, while the role of psychiatrists was recognized by only 27.2%. Regarding their own perceptions of education, the majority of respondents (213, 70.8%) do not feel that they have been adequately trained about NM and its applications to medicine.

**Table 2:** Medical students' knowledge regarding NM devices NM: neuromodulation, FDA: Food and Drug Administration

Knowledge (n=301)			
Question	Yes (%)	No (%)	I don't know (%)
Is NM an FDA-approved treatment?	37.5	5.0	57.5
Is NM useful in treating psychiatric disorders?	37.9	11.3	50.8
Is NM useful in treating movement disorders?	43.2	8.0	48.8
Do the effects of NM last only a short while?	15.3	16.6	68.1
Does NM result in a permanent cure?	14.0	23.3	62.8
Are psychiatrists involved in administering NM?	27.2	13.6	59.1
Are neurologists involved in administering NM?	43.5	11.0	45.5
Are neurosurgeons involved in administering NM?	41.2	10.6	48.2
Does NM often worsen or improve the psychiatric illness?	19.6	10.6	69.8

**Table 3:** Medical students' self-perception of education regarding NM devices NM: neuromodulation

Self-perception of education (n=301)			
Do you feel you have been trained about NM appropriately and its applications to medicine?	Yes (%)	No (%)	Somewhat (%)
	8.0	70.8	21.3

**Table 4:** Attitudes of medical students toward NM devices NM: neuromodulation

Attitude (n=301)					
Question	Strongly agree (%)	Agree (%)	Neither agree nor disagree (%)	Disagree (%)	Strongly disagree (%)
The procedure is associated with severe adverse effects	9.3	18.6	62.1	9.3	0.7
The procedure is associated with brain damage	4.0	19.3	58.1	15.9	2.7
NM is a painful procedure	8.6	15.6	59.5	13.0	3.3
NM is dangerous and should not be used	5.0	11.6	49.5	26.6	7.3
I would advise a close relative to receive NM if recommended	11.3	23.6	58.5	4.7	2.0
If required, I would undergo NM	13.6	25.6	54.2	4.0	2.7
NM is often given to people who do not need it	5.6	9.6	48.8	17.6	18.3

**Table 5:** Medical students' self-perception of knowledge regarding NM devices NM: neuromodulation

Self-perception of knowledge							
How would you rate your understanding of NM?	1 (%)	2 (%)	3 (%)	4 (%)	5 (%)	6 (%)	7 (%)
	46.2	19.3	16.3	11.0	3.7	1.7	2.0

**Figure 1:**
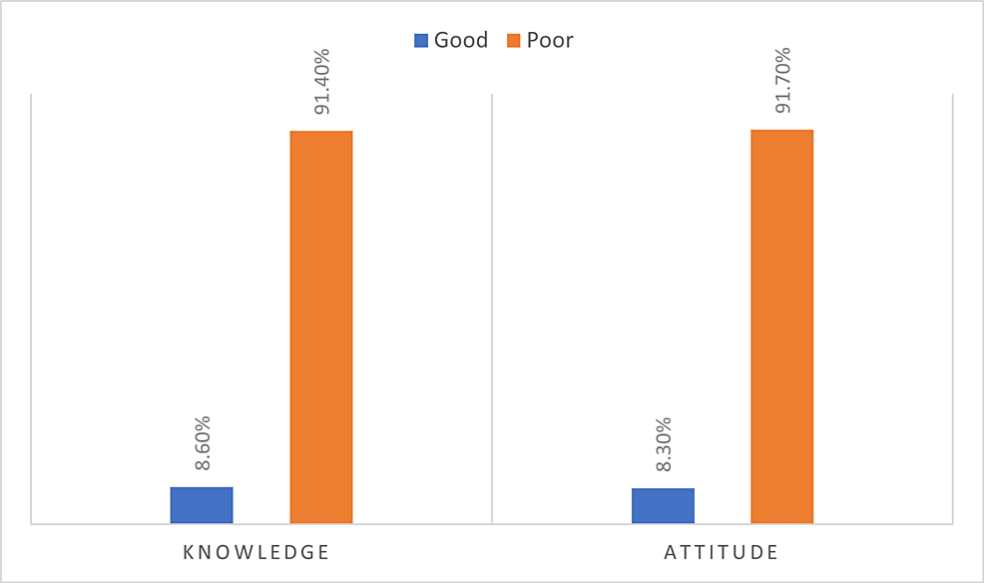
Participants' overall knowledge and attitude levels regarding NM devices

As for the attitude section, the results show that a majority of respondents did not know whether NM is associated with severe adverse effects (187, 62.1%), brain damage (175, 58.1%), or pain (179, 59.5%). Furthermore, a significant proportion of respondents were not sure whether NM is dangerous and should not be used (149, 49.5%), whether they would advise a close relative to receive NM (176, 58.5%), or whether they would undergo NM if required (163, 54.2%). Additionally, the majority were not sure if NM is often given to people who do not need it (147, 48.8%). Finally, in the self-perception of knowledge section, respondents rate their understanding of NM on a scale of one to seven, where seven is the highest. The results show that most respondents (139, 46.2%) rated their understanding of NM as one, followed by two (58, 19.3%), three (49, 16.3%), and four (33, 11.0%), and only a minority (22, 7.4%) rated their understanding to be five or higher. Overall levels of knowledge and attitude are shown in Figure 1.

Table 6 shows the association between different demographics and the level of knowledge. Regarding the academic year, although the p-value does not show statistical significance (p=0.99), there is a high prevalence of poor knowledge among students in both the pre-clinical and clinical years (116, 91.3% and 159, 91.4%, respectively). The intended specialty had an influence on the knowledge level (p=0.01), with emergency medicine and neurology/psychiatry among the specialties that recorded a higher level of knowledge compared to other specialties. In addition, upon asking participants whether they had a family member who had been treated with an NM device, 10 answered "yes," and eight (80%) of them had poor levels of knowledge, while 240 answered "no", 224 (93.3%) of them had poor knowledge, and for the remaining 51 participants who did not know, 43 (84.3%) had poor knowledge, showing a significant association (p=0.049).

**Table 6:** Association of sociodemographic factors with the total level of knowledge regarding NM devices among medical students *P-value <0.05 is statistically significant

Factors		Knowledge level			
Gender		Good	Poor	Total	P
Female	n (%)	20 (10.2)	176 (89.8)	196 (100)	0.186
Male	n (%)	6 (5.7)	99 (94.3)	105	
Academic year		Good	Poor	Total	P
Clinical	n (%)	15 (8.6)	159 (91.4)	174 (100)	0.99
Pre-clinical	n (%)	11 (8.7)	116 (91.3)	127 (100)	
Intended specialty		Good	Poor	Total	P
​Internal medicine	n (%)	4 (8.3)	44 (91.7)	48 (100)	0.01*
​Medical sub-specialty	n (%)	4 (12.9)	27 (87.1)	31 (100)	
​Neurology/psychiatry	n (%)	4 (16.0)	21 (84.0)	25 (100)	
​Undecided	n (%)	5 (5.3)	89 (94.7)	94 (100)	
Emergency medicine	n (%)	4 (21.1)	15 (78.9)	19 (100)	
General surgery	n (%)	2 (4.9)	39 (95.1)	41 (100)	
Obstetrics and gynecology	n (%)	1 (10.0)	9 (90.0)	10 (100)	
Surgical sub-specialty	n (%)	2 (6.0)	31 (94.0)	33 (100)	
Do you have a family member who had been treated with an NM device?		Good	Poor	Total	P
I don't know	n (%)	8 (15.7)	43 (84.3)	51 (100)	0.049*
No	n (%)	16 (6.7)	224 (93.3)	240 (100)	
Yes	n (%)	2 (20.0)	8 (80.0)	10 (100)	

Table 7 demonstrates the association between demographics and attitude levels. Out of a total of 196 females, only eight (4.1%) had a good attitude toward NM, while 188 (95.9%) had a poor attitude. Among the 105 males, 17 (16.2%) had a good attitude toward NM, while 88 (83.8%) had a poor attitude. The statistical significance of the difference in attitude between males and females had a very low p-value (p=0.0003), suggesting a strong association with a larger proportion of females having a poor attitude toward NM compared to males. Interestingly, preclinical students had a better level of knowledge (11.0%) compared to clinical students (6.3%). A significant association was noted between the level of attitude and having a family member who had been treated with an NM device (p=0.04).

**Table 7:** Association of attitudes with the total level of knowledge regarding NM devices among medical students *p-value <0.05 is statistically significant **p-value <0.01 is statistically highly significant

Factors		Attitude level			
Gender		Good attitude	Poor attitude	Total	P
Female	n (%)	8 (4.1)	188 (95.9)	196 (100)	0.0003**
Male	n (%)	17 (16.2)	88 (83.8)	105 (100)	
Academic year		Good attitude	Poor attitude	Total	P
Clinical	n (%)	11 (6.3)	163 (93.7)	174 (100)	0.54
Pre-clinical	n (%)	14 (11.0)	113 (89.0)	127 (100)	
Intended specialty		Good attitude	Poor attitude	Total	P
​Internal medicine	n (%)	5 (10.4)	43 (89.6)	48 (100)	0.94
​Medical sub-specialty	n (%)	1 (3.2)	30 (96.8)	31 (100)	
​Neurology/psychiatry	n (%)	2 (8.0)	23 (92.0)	25 (100)	
​Undecided	n (%)	4 (4.3)	90 (95.7)	94 (100)	
Emergency medicine	n (%)	2 (10.5)	17 (89.5)	19 (100)	
General surgery	n (%)	7 (17.1)	34 (82.9)	41 (100)	
Obstetrics and gynecology	n (%)	1 (10.0)	9 (90.0)	10 (100)	
Surgical sub-specialty	n (%)	3 (9.1)	30 (90.9)	33 (100)	
Do you have a family member who had been treated with an NM device?		Good attitude	Poor attitude	Total	P
I don't know	n (%)	4 (7.8)	47 (92.2)	51 (100)	0.04*
No	n (%)	18 (7.5)	222 (92.5)	240 (100)	
Yes	n (%)	3 (30.0)	7 (70.0)	10 (100)	

**Figure 2:**
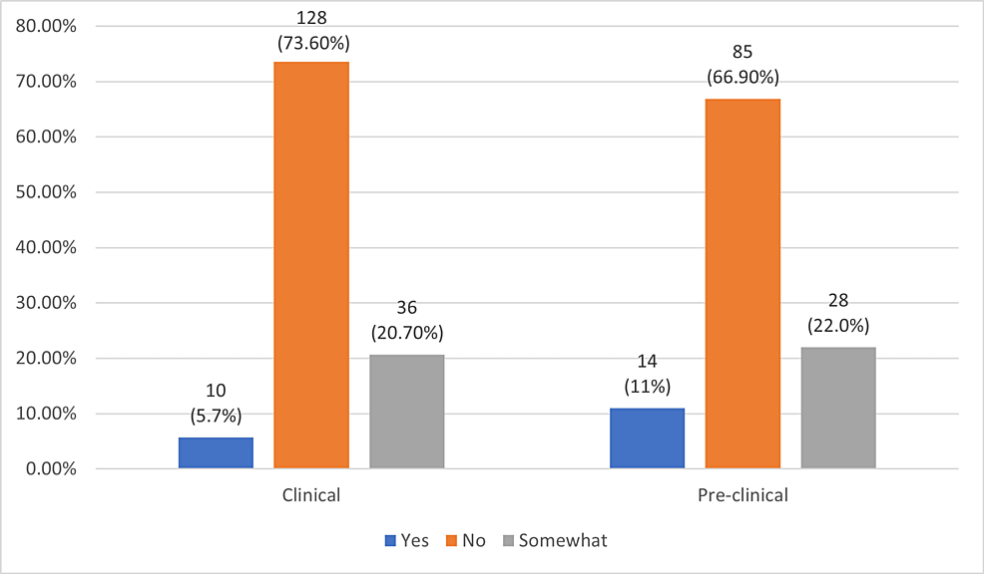
Association of self-perception of education regarding NM among medical students with their academic year

Figure 2 demonstrates the disruption of students’ answers to the question, "Do you feel you have been trained appropriately about NM and its applications to medicine?" Despite the absence of a significant association between clinical and pre-clinical students (P=0.214), pre-clinical students showed more education satisfaction (11%) when compared to clinical students (5.7%).

## Discussion

The current study explored the medical students' knowledge and perception of NM as a treatment modality, and a comparison was made between students in their clinical years and those in their pre-clinical years. Regardless of whether students were in the pre-clinical or clinical stages of their education, the study found that there was a significant lack of knowledge regarding the practical use of NM among the participants.

The initial assumption was that students who had more exposure to clinical settings would have higher levels of confidence and knowledge. However, the study's results contradicted this assumption. This contradiction could be indicative of both a lack of basic understanding in the classroom and insufficient practical training in NM within clinical placements.

Considering the increasing use of NM therapy, it's unlikely that physicians who specialize outside of psychiatry, neurology, or neurosurgery would have a strong understanding of the complex details, nuances, and advanced physiological principles behind NM devices. However, due to the growing popularity of this treatment, these physicians might come across patients who either need NM therapy or already have a stimulator in place. Therefore, it's important for all medical practitioners to have a basic understanding of the rationale and indications of NM devices. This knowledge is crucial for providing the best possible care for patients. However, our findings show that a significant majority of the medical students surveyed (70%) were able to identify an inadequacy of their education regarding this therapeutic approach, which is consistent with a previous study of DBS [12].

This situation highlights a significant gap in medical understanding, which is both concerning and enlightening. Even though psychiatry and neurology are major topics in medical education, it seems that NM is not adequately addressed in the current medical curriculum. It's worth noting that while 70% of students were not satisfied with the information available about NM, an unexpected 34.9% of students still felt confident in recommending NM procedures for their family members. This occurrence might potentially showcase the confidence that medical students maintain in their chosen career paths despite lacking essential foundational knowledge.

Another worrying finding is the fact that most students (57.5%) lacked awareness about the FDA-approved status of NM as a therapeutic technique, even though it has been proven effective in treating epilepsy, motor impairments, and psychiatric disorders. Additionally, it's important to mention that while 37.9% of students were aware of NM's effectiveness in treating psychiatric issues, a significant portion (62.1%) were either unsure or doubtful about psychiatrists being involved in administering DBS. Importantly, a smaller proportion of students (37.9%) believed in the potential of NM devices for alleviating psychiatric disorders. This observation is consistent with findings from a study conducted in Germany [9].

Given its current indication for treating obsessive-compulsive disorder and its potential use in other psychiatric disorders [8], it's crucial to address this lack of knowledge. Doing so will help future medical professionals use available treatments effectively, including NM. Regarding attitudes and biases, a small percentage of students (34.9%) expressed their readiness to suggest NM to a family member, while 39.2% were open to undergoing NM themselves if necessary. Interestingly, this differs from the results of Virginia Tech Carilion School of Medicine, which showed an overall positive attitude with prevalence rates of 65% and 74%, respectively [12].

Moreover, our findings have shown that a considerable number (33.9%) of students believed that NM is not inherently dangerous and should be considered a viable treatment option. At the same time, a larger proportion (62.1%) were unsure about whether NM could lead to serious side effects or brain damage.

The bias inventory has shown positive results overall. Thus, it's important to highlight that the lack of sufficient information resources and perceived lack of guidance about NM among medical students aren't primarily due to a negative bias against this field. Instead, they stem from a fundamental difficulty in fully understanding the subject. This finding emphasizes the idea that including NM in the current medical education curriculum would be welcomed enthusiastically, as it has the potential to improve patient outcomes.

The knowledge, attitudes, and assumptions held by physicians wield a substantial influence over patients' choices concerning medical-surgical interventions. Consequently, the identified gaps in understanding revealed in this study highlight the need for improvements in medical education and upcoming residency training programs. These developments aim to improve physicians' comprehension of NM therapy and patient outcomes.

To address the observed knowledge and confidence gaps among medical students in relation to NM therapies, a set of comprehensive recommendations is proposed. These include enhancing medical curricula to incorporate theoretical and practical NM education, fostering collaboration with relevant specialties, prioritizing continuing medical education for NM advancements, conducting public awareness initiatives, encouraging student involvement in NM research, ensuring regular updates to curriculum content, and establishing networking and mentorship opportunities. These measures collectively aim to equip future medical professionals with the necessary skills and knowledge to navigate NM therapies effectively, ensuring optimal patient outcomes and ethical decision-making.

Limitations

This study had some limitations, despite efforts to ensure accurate and representative outcomes. The use of an online questionnaire and a predominantly female respondent (65.1%) may have introduced unintended biases. It is important to consider these limitations when interpreting the results. Additionally, we adapted a previously established questionnaire for our study, making the necessary modifications for our context. However, it's important to note that, as pointed out in the original paper for this questionnaire, a standardized validation metric has not yet been developed. Nonetheless, the study provides valuable insights into the knowledge and perception of UQU medical students about NM devices and aligns with previous research. Further research is needed with diverse methodologies, exploring different sociodemographic characteristics and unexplored universities in Saudi Arabia.

## Conclusions

NM is an effective treatment for several neurological disorders, including Parkinson’s disease, essential tremor, dystonia, and epilepsy. As a result of the advances in research, both the number of patients being treated with this therapy and the number of physicians seeing these patients have increased. An overall lack of knowledge of NM devices was observed among medical students in this study. Hence, it is essential that all students be educated at least on the basic principles and indications for this therapy to treat patients appropriately. Other studies on the knowledge gained on this topic in other countries with different educational systems would be valuable, but unfortunately, such studies are not available. Further, it may be helpful to conduct future surveys to better understand why NM knowledge is still limited and how this could be improved not only among medical students but also among general practitioners, neurologists, and psychiatrists. To improve the knowledge level in this field, the teaching of NM in medical schools may take various forms, including lectures, video or live observation, and in some cases, direct participation under supervision.
